# Meta Analysis of Methylenetetrahydrofolate Reductase (MTHFR) *C677T* polymorphism and its association with folate and colorectal cancer

**DOI:** 10.1186/s12885-025-13546-w

**Published:** 2025-01-29

**Authors:** Meng Ye, Guojie Xu, Liming Zhang, Zhihui Kong, Zhenhua Qiu

**Affiliations:** 1 The First Clinical Medical College, Guangdong Medical University, Zhanjiang, 524023 P.R. China; 2 Cancer Center, Union Hospital, Tongji Medical College, Huazhong University of Science and Technology, Wuhan, 430022 P.R. China; 3 Institute of Radiation Oncology, Union Hospital, Tongji Medical College, Huazhong University of Science and Technology, Wuhan, 430022 P.R. China; 4 Department of Laboratory Medicine, Jingshan People’s Hospital, Jingshan, 431800 P.R. China; 5 Department of Laboratory Medicine, Affiliated Gaozhou People’s Hospital, Guangdong Medical University, Maoming, 525200 P.R. China

**Keywords:** Colorectal cancer, MTHFR, Folate, Meta-analysis

## Abstract

**Background:**

DNA hypomethylation and uracil misincorporation into DNA, both of which have a very important correlation with colorectal carcinogenesis. Folate plays a crucial role in DNA synthesis, acting as a coenzyme in one-carbon metabolism, which involves the synthesis of purines, pyrimidines, and methyl groups. MTHFR, a key enzyme in folate metabolism, has been widely studied in relation to neural tube defects and hypertension, but its role in colorectal cancer remains underexplored. Therefore, understanding the role of folate and MTHFR genes in colorectal cancer may be helpful for potential preventive or therapeutic interventions. In this meta-analysis, the effects of MTHFR genotype and folate intake on colorectal cancer incidence were analyzed.

**Methods:**

We searched PubMed,Embase, Web of Science, and CNKI database to identify relevant studies up to January 2024. We included a series of studies on the association of *MTHFR C677T* genotype and folate intake with colorectal cancer incidence. The meta-analysis was conducted in accordance with the Preferred Reporting Items for Systematic Reviews and Meta-Analyses Protocols (PRISMA-P). It included 100 studies (39702 cases and 55718 controls),that investigated the association between the *MTHFR* C677T polymorphism and colorectal cancer (CRC). Additionally, the analysis incorporated further stratification by ethnic population and geographical region. Furthermore, Six of the studies which clarified high amount of folate might be a protective factor for CRC in all three *MTHFR C677T* genotype, especially in *TT* genotype.

**Results:**

*MTHFR 677TT* genotype was negatively associated with CRC incidence compared with CC genotype (OR = 0.89; 95% CI: 0.85–0.93; *P* < 0.00001; Z = 5.17). *MTHFR 677CT* genotype was not significantly associated with colorectal cancer incidence (OR = 1.00; 95% CI: 0.98,1.03). A negative correlation between TT genotype and CRC was observed in ethnics of Asians (OR = 0.83, 95% CI: 0.76, 0.91), Caucasians (OR = 0.93, 95% CI: 0.88, 0.99) and the region of USA (OR = 0.77, 95% CI: 0.71, 0.85), Asia (OR = 0.93, 95% CI: 0.86, 1.00) and Europe (OR = 0.93, 95% CI:0.87, 1.00),but not in Indian (TT: OR = 1.67, 95% CI: 1.06, 2.63; CT: OR = 1.31, 95% CI: 1.00, 1.73)). Amount folate intakes might reduce the morbidity of CRC for people in *MTHFR 677TT* genotype (OR = 0.68; 95% CI: 0.48,0.96; *P* = 0.03).

**Conclusion:**

The analysis showed that the incidence of colorectal cancer was reduced among individuals with* TT* genotype. The individuals with *TT* genotype and amount folate intake may collectively improve the incidence of colorectal cancer. While the *MTHFR 677TT* genotype is associated with a reduced risk of CRC, especially in certain populations, these findings should be interpreted with caution due to the limitations of retrospective studies.

**Supplementary Information:**

The online version contains supplementary material available at 10.1186/s12885-025-13546-w.

## Introduction

### Folate and its metabolism

**Fig. 1:**
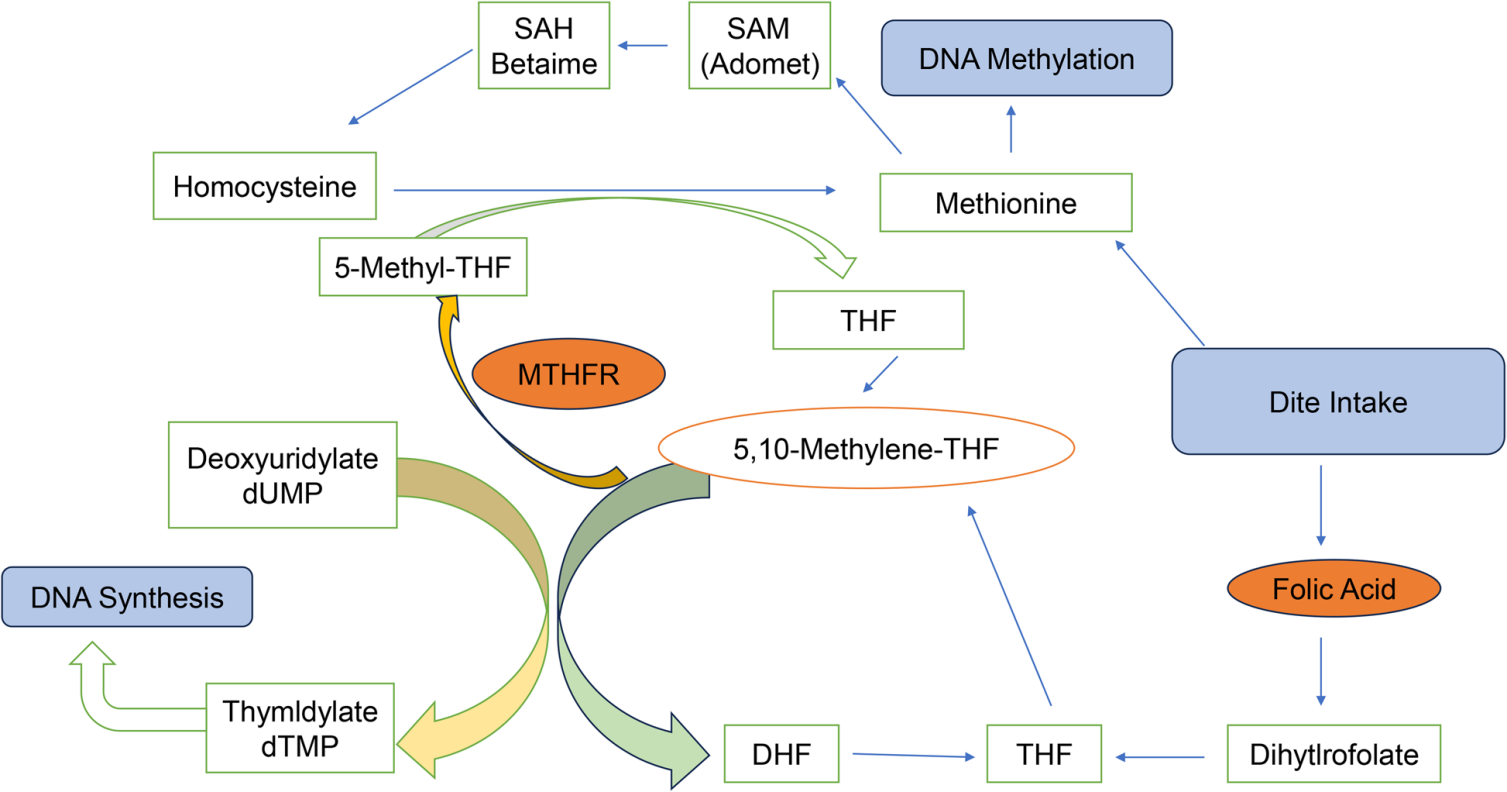
The schematic figure of folate metabolism

Folate, also known as vitamin B9, is one of the water-soluble B vitamins [1]. Folate is first hydrolyzed to 5-methyltetrahydrofolate in the jejunum, and methyltetrahydrofolate enters the cell under the action of the cell membrane reduced folate carrier-1 (RFC-1) for intracellular folate cycle metabolism. As shown in Fig. 1, 5-Methylenetetrahydrofolate is the most common form of folate in the body, and it is converted to 5-methylenetetrahydrofolate (5-MTHF). Under the joint action of methionine synthase (MTR) and methionine synthesis reductase (MTRR), 5-MTHF synthesizes methionine (Met) with homocysteine (Hcy), which enters the methionine cycle and provides a one-carbon unit for intracellular methylation metabolism [2, 3]. Therefore, when folate intake is reduced, intracellular methylation levels are also reduced, which induces a range of diseases [4, 5]. One in vitro and in vivo experiments have also confirmed that low folate increases the risk of CRC [6].

### MTHFR

MTHFR is a key enzyme in human homocysteine and folate metabolism. The gene encoding MTHFR is located on human chromosome 1p36.3, encodes 656 amino acids, has a relative molecular mass of 45, and a total genome length of 15,835 bp, including 11 exons and 10 introns, with exon lengths ranging from 99 to 252 bp and intron lengths of 192–981 bp [7]. MTHFR currently has up to 15 mutation sites, the most common being the 677C > T and 1298A > C mutation sites. *MTHFR 677* is in exon 4, where cytosine (C) is replaced by thymine (T), and the corresponding alanine is changed to valine. These mutations reduce MTHFR enzyme activity [7, 8]. Fifteen-point mutation types have been identified in human MTHFR, the most common and important of which is the mutation in *C677T*, which manifests itself in three genotypes, CC, CT, and TT. *MTHFR C677T* mutation can cause resulting in a decrease in heat resistance and enzyme activity.

### Colorectal cancer

Colorectal cancer (CRC) is the third most common cancer and second most common cause of cancer-related deaths worldwide [9, 10]. Colorectal carcinogenesis is a complex process. Among them, aberrant methylation of related genes is an important element in the development and progression of CRC [11, 12]. For example, mutations in proto-oncogenes often lead to colorectal cancer [13, 14]. It is widely known that folate (FA) and methylenetetrahy-drofolate reductase (MTHFR) play important roles in the process of gene methylation. In recent years, with the gradual improvement of people's living standards, eating habits have also undergone great changes, and its morbidity and mortality have shown a significant rising trend.On the one hand, folic acid plays a certain role in the occurrence and development of tumors.On the other hand, folic acid is included in the diet, and folic acid is also an important nutritional supplement absorbed through the intestines. Based on this, it is valuable to explore the relationship between folic acid and its key metabolic genes and colorectal cancer. MTHFR is a key enzyme in folate metabolism. MTHFR promotes the synthesis of folate into methylenetetrahydrofolate, which participates in the methionine cycle and gene methylation process [1, 15, 16]. Mutations in the relevant sites of MTHFR gene can result in a decrease in the enzyme activity of the MTHFR protein, leading to a deficiency of methylenetetrahydrofolate, which can eventually lead to a decrease in the degree of DNA methylation and microsatellite instability, and ultimately can induce colorectal neoplasm [2, 3, 17]. Therefore, it is necessary to explore the correlation between MTHFR gene polymorphisms and serum folate levels and CRC risk, and to verify that MTHFR gene variants are one of the genetic susceptibility factors of CRC, which can help to provide a reference for personalized diagnosis and treatment and provide a new idea and direction for targeted therapy.

## Materials and methods

### Literature search and data extraction

We performed a pooled analysis of published articles in PubMed,Embase, Web of Science, and CNKI database for studying the association between the *MTHFR C677T* variant and folate content and colorectal cancer. This study searched for relevant studies from November 1, 1996, to January 2024. The date of November 1, 1996, when the first study on MTHFR polymorphisms and colorectal cancer risk was published [1]. We first performed an extensive database retrieval. We searched using the keywords: ("Methylenetetrahydrofolate reductase" OR “MTHFR”) AND ("colorectal cancer" OR "colon cancer" OR "rectal cancer") AND"Genetic polymorphisms".

Titles and abstracts were reviewed by two independent researchers and independently reviewed by reviewers according to their inclusion criteria. Any disagreements between the first two reviewers regarding extracted data were resolved through discussion by a third reviewer. After multiple reviews of these articles, 100 articles met the relevance criteria and were selected. We contacted the authors several times to obtain any missing data. If the authors never responded to our inquiries, the missing data were not included in our statistics. When the same study was offered multiple times, we prioritized the most recent article containing the most recent information.

This study was a meta-analysis, so it did not require ethical approval from the institutional review board as well as the ethics committee.

The record of this systematic evaluation is available at inplasy.com. Our registration number is INPLASY2023110057 Our DOI number is 10.37766/inplasy2023.11.0057. We followed the Preferred Reporting Items for Systematic Evaluations and Meta-Analyses (PRISMA) statement and the Cochrane Collaboration (http://www.prisma-statement.org/) recommendations on reporting preferences.

### Laboratory test methods for MTHFR

Each of the articles we selected describes in detail the methods used for *MTHFR C677T g*enotyping. Most of these studies were reported by researchers.

### Inclusion criteria and study eligibility

**Fig. 2:**
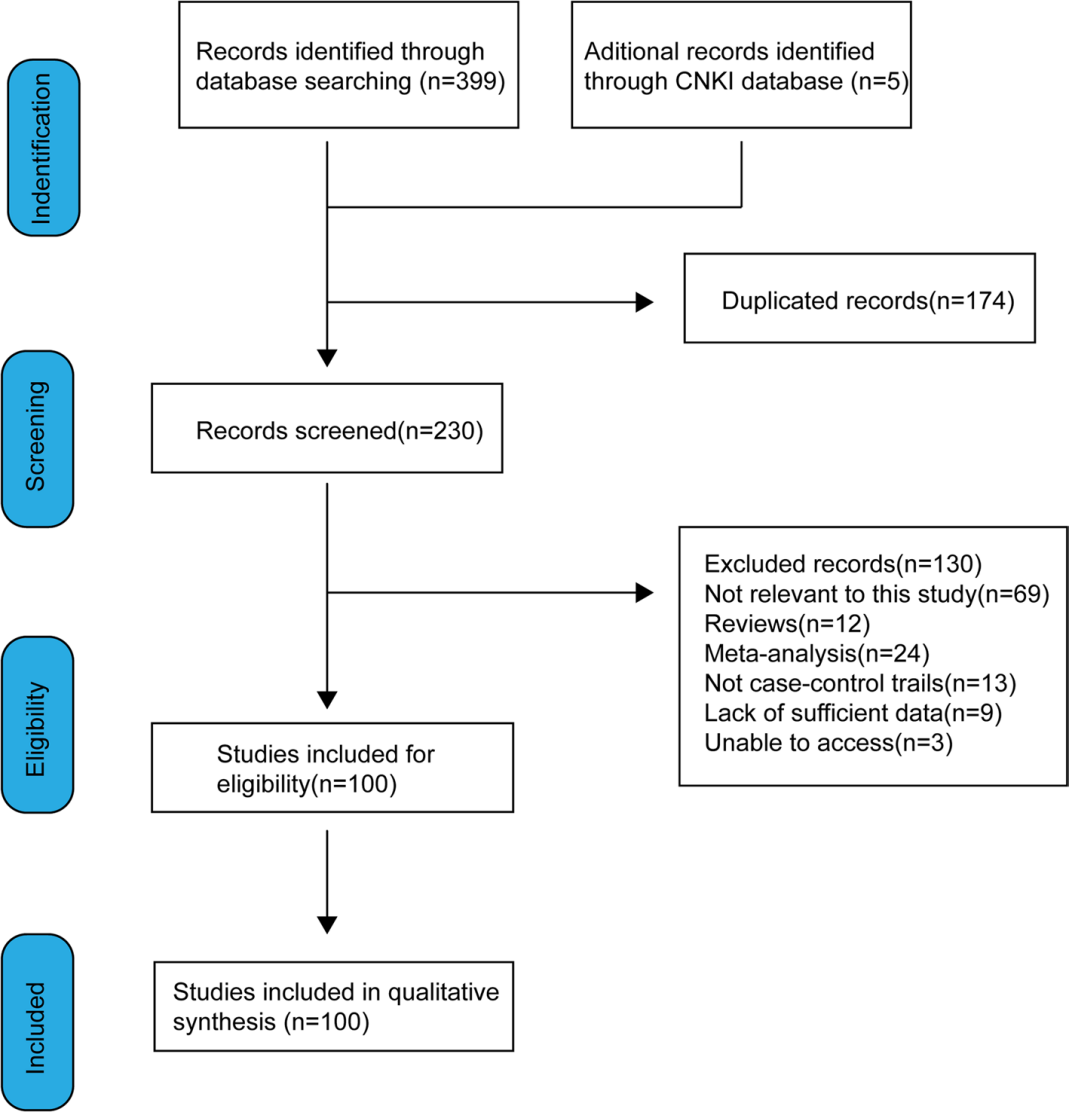
Flow chart of literature search and selection process

During the review process. Two independent researchers reviewed the titles and abstracts of 404 articles. The inclusion criteria: MTHFR genotype was conferred by using polymerase chain reaction and genomic DNA extraction from blood; case–control studies; investigating the association between *MTHFR* C677T genotype and CRC susceptibility. Two abstractors reviewed each article using a standardized data collection form and finally agreed on the articles selected. The remaining 304 articles were removed for the following reasons: they did not focus on the *677C* allele of the MTHFR gene; they did not focus on the number of cases of *CC, CT,* and *TT* genotypes in cases and controls; they were reviews or meta-analyzes; they were not case-cohort studies or retrospective analyses of cases; they had not sufficient data; or they are not relevant and focused only on the relationship between MTHFR and chemotherapy toxicity in colorectal cancer (Fig. 2).

### Quality assessment

The quality of this study was assessed using the Newcastle–Ottawa Scale, which was conducted independently by two reviewers [18]. The Newcastle–Ottawa Scale consists of three main assessment categories: Selection (adequate definition of cases, representativeness of cases, selection of controls, definition of controls), Comparability (control for important factor or additional factor), and Exposure (ascertainment of exposure, same method of ascertainment for cases and rate controls, Non-Response rate). Studies can score up to 9 points. A final score of 7 or higher indicates that the study was of high quality (Supplementary Table 1).

### Statistical analysis

The number of case groups with MTHFR genotypes (*CC, CT,* and *TT* genotypes) and the number of controls were extracted from our pooled studies. The corresponding study-specific crude advantage ratios and 95% confidence intervals for CRC risk were calculated.

We imported the above data into Review Manager version 5.4 and then calculated pooled estimates of the odds of a *TT* polymorphism occurring with a *CC* polymorphism and the odds of a *CT* polymorphism occurring with a *CC* polymorphism. Inter-study heterogeneity was assessed using chi-square test and I^2^ tests. Statistically significant heterogeneity between trials was indicated using the Cochran Q statistic *P* < 0.05 or the I^2^ statistic > 50% [19]. Depending on the degree of heterogeneity, summary statistics were calculated using either a fixed-effects model or a random-effects model. Fixed-effects models were used for initial analyses, and random-effects models were used for validation analyses if significant heterogeneity existed. The odds ratio and its 95% confidence interval were calculated to assess the association between the *MTHFR C677T* polymorphism and CRC.

Inter-study heterogeneity in our meta-analysis was assessed using forest-like plots to explore the association of folate and MTHFR genotypes with CRC development. We used the I^2^ test and chi-square test to formally check for the presence of heterogeneity. In this case, heterogeneity was categorized as low, moderate, or high for I^2^ values of 25%, 50%, and 75%, respectively, and the chi-square test was considered statistically significant with a *P* value of < 0.05. Pooled risk estimates were calculated using a random-effects model because the true effect sizes may not be constant across all included studies and the effect measures were odds ratios (ORs).

Publication bias was assessed using funnel plots, followed by the regression-based approach proposed by Egger et al. and the rank correlation test provided by Begg and Mazumdar, which is a formal and objective test for publication bias [20].

## Results

### Statistical analysis for meta-analysis

The meta-analysis included 100 studies with a total of 95420 subjects (39702 cases and 55718 controls). Table 1 provides the general characteristics of the studies. In these studies, For the association of CRC with *MTHFR CT* and *TT* genotypes, the Q-test for homogeneity was not significant; therefore, the fixed-effects pool method was used. And the random effects pool methods was used to verify it (showed in Supplementary Figure).

**Table 1:**
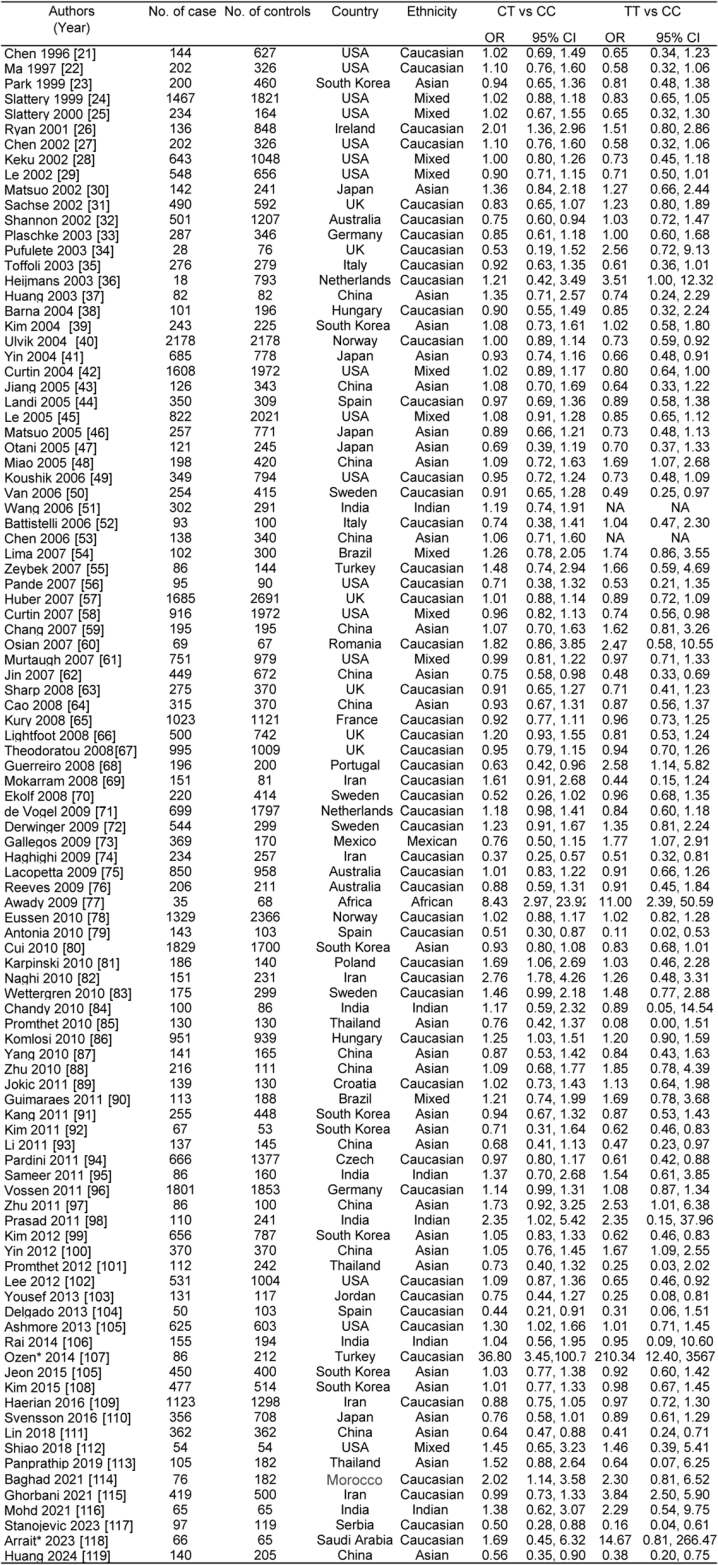
Relation between *MTHFR C677T* genotype and colorectal cancer: general characteristics of studies included in a meta-analysis [21–119]

*Abbreviations*: *CI* confidence interval, *NA* not applicable, *OR* odds ratio

Ozen* and Arrait*: the case of controls of MTHFR 677T was zero

As assessed by Begg's plot and Egger's test, publication bias was not significant [20]. For both ratio ratios, there were no studies that unduly influenced the pooled estimates.

**Fig. 3:**
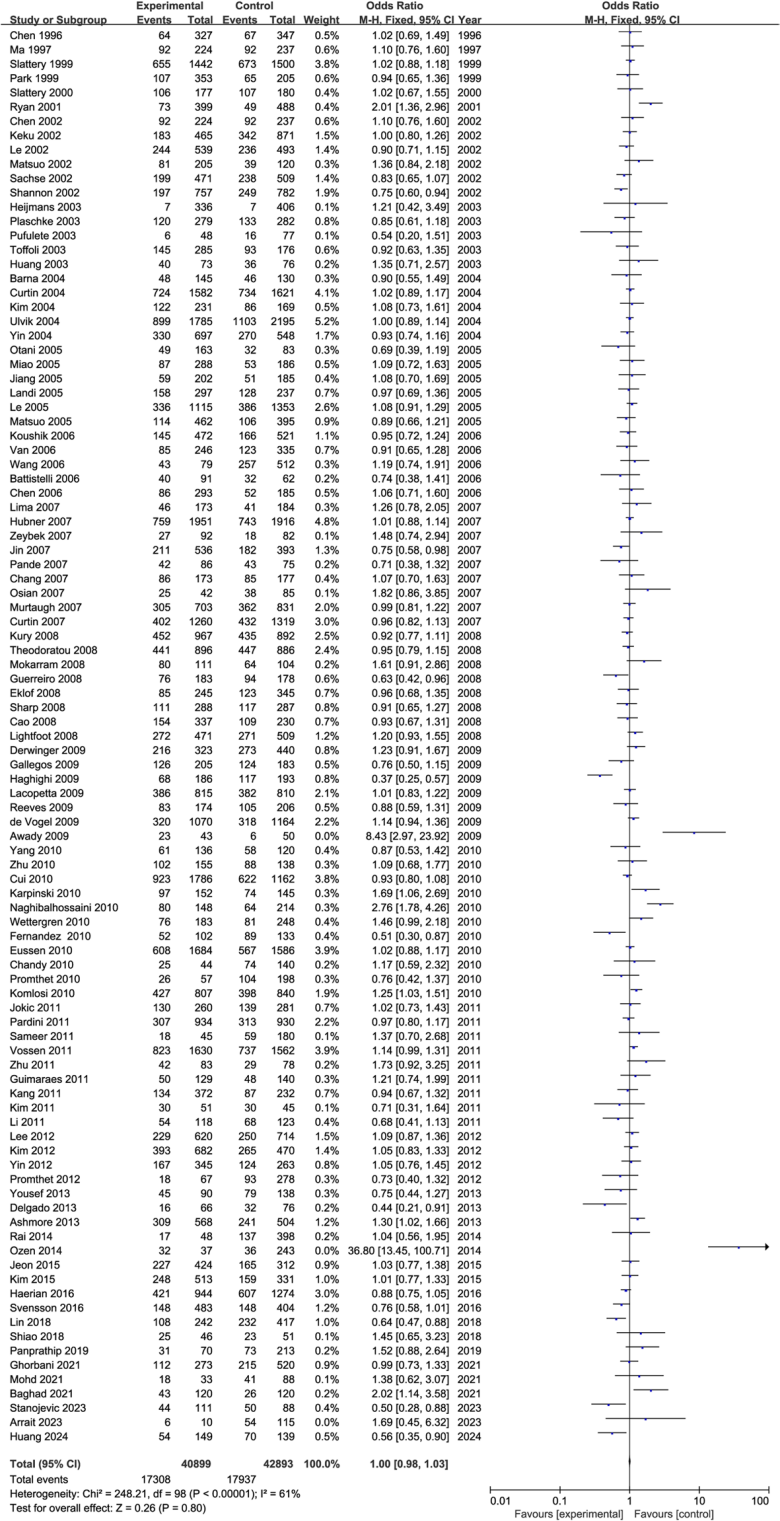
The forest plot of meta-analysis odds ratios for colorectal cancer among persons with the *MTHFR 677CT* and *677CC* genotype

The overall meta-analytic superiority ratio for CRC in patients with the *MTHFR 677CT* genotype was 1.00 (95% CI: 0.98, 1.03) (Fig. 3 and Supplementary Fig. 1). The 95% confidence interval for the superiority ratio of the 100 studies contained 1, of which 53 showed a positive correlation, 45 showed a negative correlation, and 2 were equal to 1. Therefore, there was no statistical correlation between *MTHFR CT* genotype and CRC incidence.

**Fig. 4:**
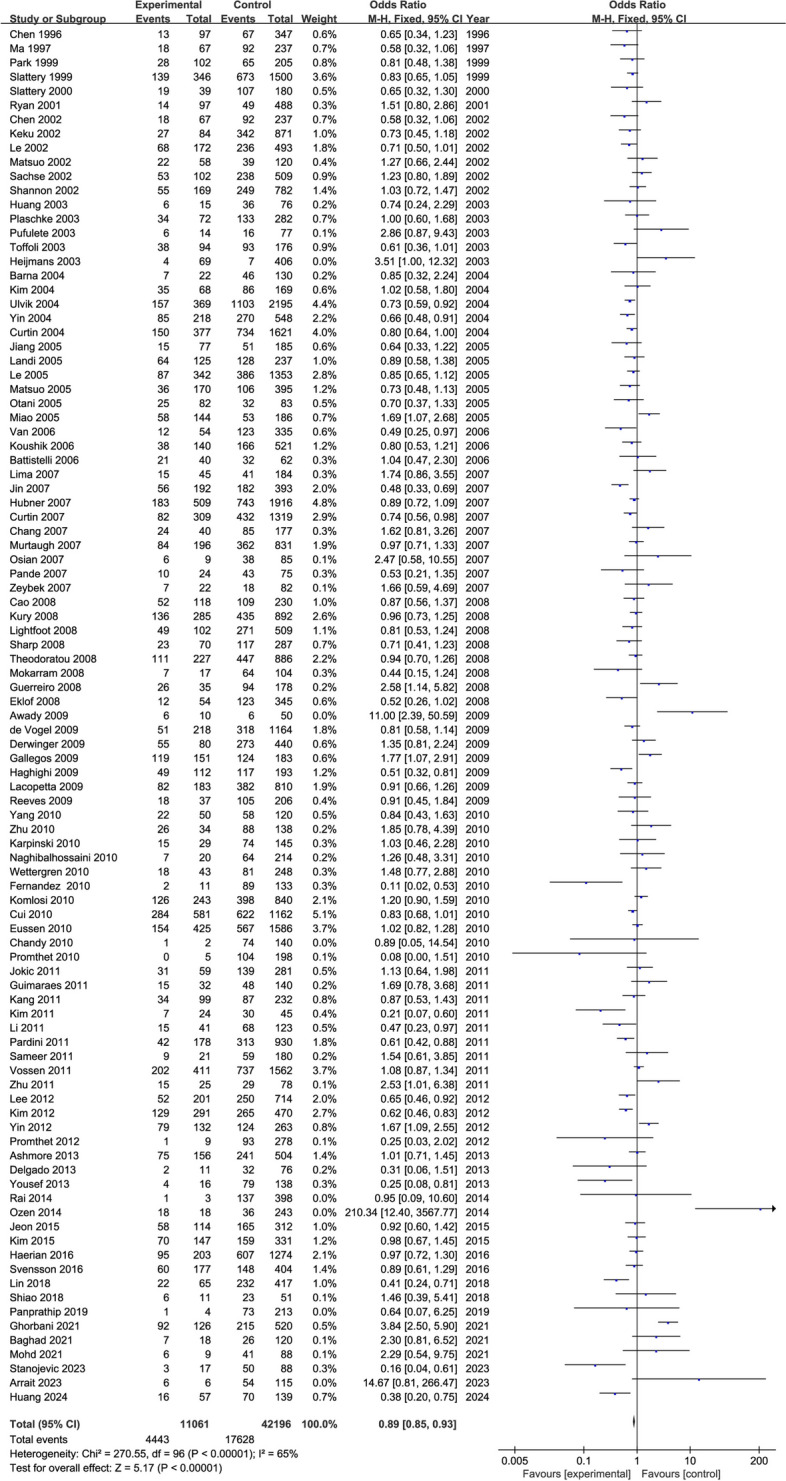
The forest plot of meta-analysis odds ratios for colorectal cancer among persons with the *MTHFR 677TT* and *677CC* genotype

The overall meta-analysis of *MTHFR 677TT* genotypes had a dominance ratio of 0.89 (95% CI: 0.85, 0.93, *P* < 0.00001 and a Z = 5.17) (Fig. 4 and Supplementary Fig. 2). Thirty seven out of 98 studies had dominance ratios that indicated a positive correlation, 60 had dominance ratios that indicated a negative correlation, two no data, and one had a dominance ratio of 1.00. This result suggests that *TT* genotype is inversely related to the incidence of CRC.

### Colorectal cancer and MTHFR C677T in racial/geographic variants

Of the above studies, we found 51 articles reporting data on Caucasians, 30 on Asians, and 6 on Indians. (Table 2 and Supplementary Table 2). There was an inverse association between *MTHFR 677TT* genotype and CRC that was statistically. The specific data is that OR of Caucasians is 0.93 (95% CI: 0.88, 0.99) and OR of Asians is 0.83 (95% CI: 0.76, 0.91). There was no significant difference between Caucasians and Asians in *MTHFR 677CT* genotype. However, an inverse association was observed in Indian (TT: OR = 1.67, 95% CI: 1.06, 2.63; CT: OR = 1.31, 95% CI: 1.00, 1.73). Moreover, in these studies, we analyzed the data by geographic region (United States, Europe, Asia, etc.) and observed a consistent negative association between the *MTHFR 677TT* genotype and CRC in the United States (OR:0.77, 95% CI:0.71, 0.85), Asia (OR = 0.93, 95% CI: 0.86, 1.00) and Europe (OR = 0.93, 95% CI:0.87, 1.00).

**Table 2:**
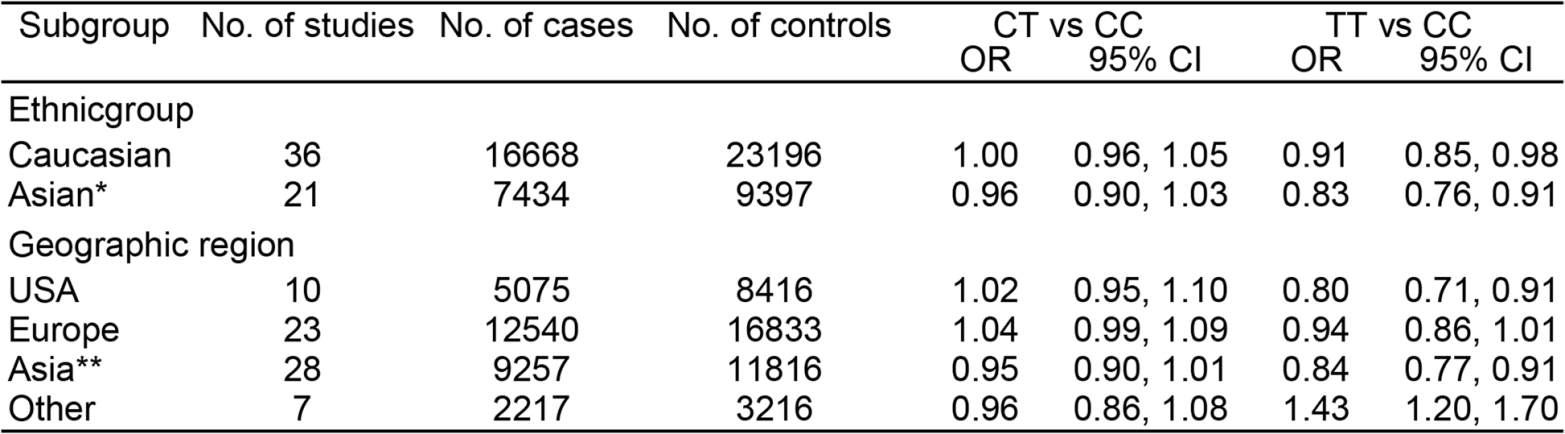
Meta-analysis odds ratios for the relation between the genotype of *MTHFR 677TT* and colorectal cancer, by Ethnic Group and Geographic Region

Abbreviations: CI confidence interval, OR odds ratio

^a^Meta-estimate *P* value: Caucasian-CT, *P*=0.88; TT, *P*=0.01; Asian-CT, *P* =0.22; TT, *P*<0.0001; USA-CT, *P*=0.58; TT; *P* =0.0003; Europe-CT, *P*= 0.16; TT, *P*=0.11; Asia-CT, *P*=0.08; TT, *P*<0.0001; Other-CT, *P*=0.49; TT, *P*<0.0001

Asian*: the study of Wang et al and Sameer et al were not included in the Asian ethnicgroup analysis because of targeting Indian

Asia**: the study of Wang et al and Sameer et al were included in the Asia geographic group analysis

### Folate differences in colorectal cancer and MTHFR C677T

Folate is a key coenzyme for DNA synthesis and DNA methylation [120, 121]. Several studies have found that folate deficiency increases the risk of malignant tumors, especially colorectal cancer [120, 122]. The effect of folate intake was investigated from the 100 articles that have been screened for the association of the MTHFR gene with colorectal cancer by screening. The articles based on the following criteria: Each gradient of folate intake had a specific number of cases for a particular genotype of *MTHFR C677T* for both the CRC patients and the control group (or data is available for calculation) for a dose–response analysis.

**Fig. 5:**
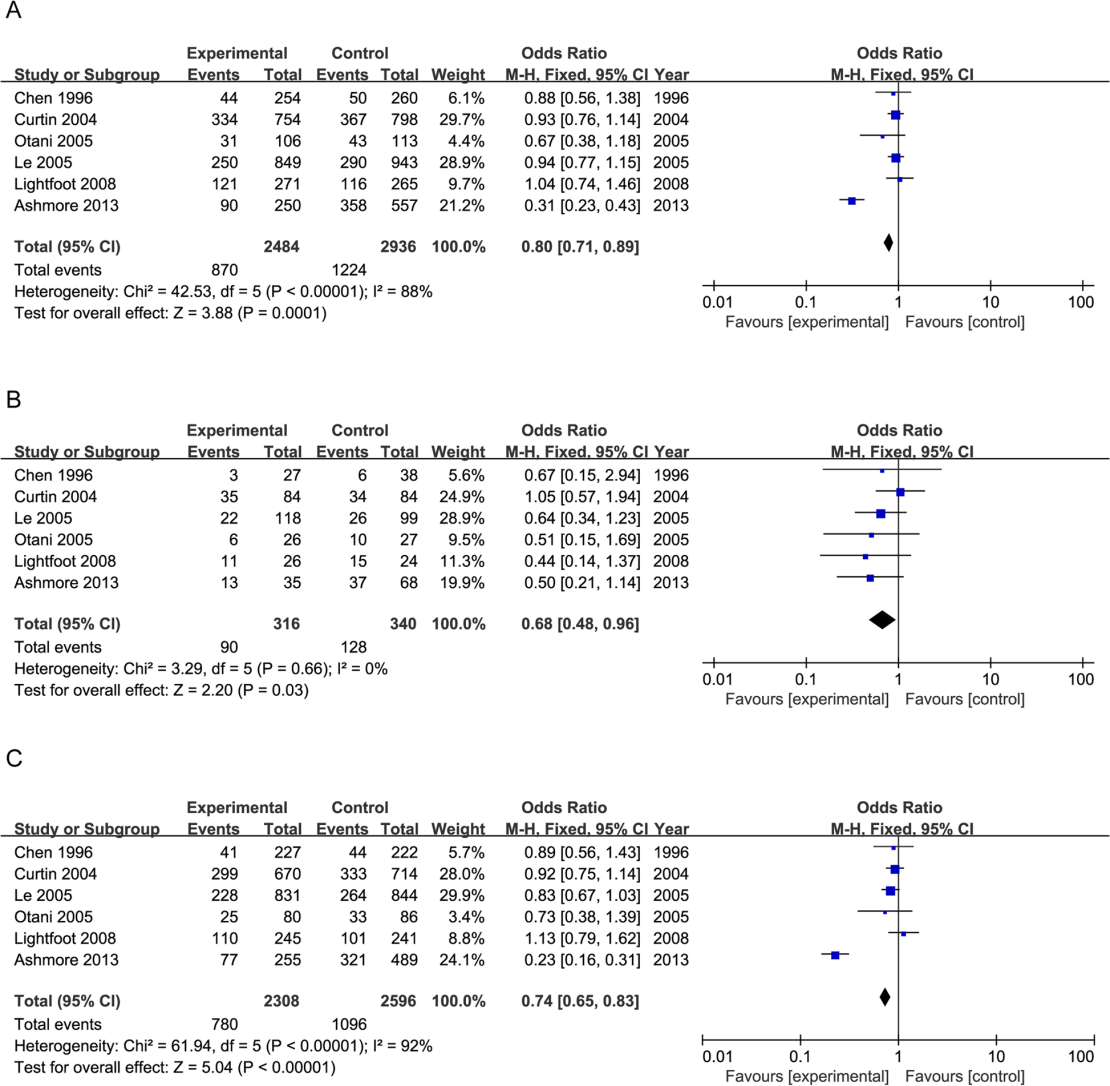
The forest plot of meta-analysis odds ratios for colorectal cancer among persons with the *MTHFR 677 T* genotype with the enhanced intake of folate. The patients were divided into two groups: high and low intakes of folate. The high group was more than the low group at least 100 μg/day. **A** The forest plot of meta-analysis of the association between CRC susceptibility and intake of folate. **B** The forest plot of meta-analysis of the association between CRC susceptibility and intake of folate among the *MTHFR 677TT* genotype. **C** The forest plot of meta-analysis of the association between CRC susceptibility and intake of folate among the *MTHFR 677CC* + *CT* genotype

We screened 6 articles from by criteria that included folate data and met the criteria above. We calculated an overall meta-analytic superiority ratio of 0.80 (95% CI: 0.71, 0.89) for CRC susceptibility with folate intakes (Fig. 5A). But verified in random effects model, the value was 0.75 (95% CI: 0.52, 1.07) (Supplementary Fig. 3A). One item showed a positive correlation and 5 showed a negative correlation. It clarified that folate intakes might be a protective factor to CRC.

Moreover, we analyzed the association between CRC susceptibility and intake of folate among the *MTHFR C677T* genotype. The overall meta-analytic superiority ratio for patients with the TT genotype was 0.68 (95% CI: 0.48, 0.96, P = 0.03) in fixed effect model (Fig. 5B) and was 0.67 (95% CI: 0.47, 0.95, P = 0.02) in random effect model (Supplementary Fig. 3B).The outcome of CT + CC genotype in fixed effect model was 0.74 (95% CI: 0.65, 0.83) (Fig. 5C). An inversed outcome verified in random effect model showed CT + CC genotype was 0.71 (95% CI: 0.45, 1.12) (Supplementary Fig. 3C). Our previous study yielded there might be a negative correlation between *MTHFR 677TT* and CRC. Our results showed the incidence of colorectal cancer might be lower in TT genotypes when the intake of folate enhanced.

## Discussion

### Overall association of MTHFR C677T genotype with colorectal cancer risk

Up to now, research on colorectal cancer risk, MTHFR, and folic acid intake has led to greater uncertainty in various conclusions due to many differences in experimental design, sample type, stratification, race, geography, and dietary habits. Therefore, meta-analysis is necessary, which can combine a large number of studies that meet the requirements to maximize the accuracy of the conclusions.

The results of the current meta-analysis confirm that the *MTHFR 677TT* polymorphism may be associated with reduced CRC risk, a finding that is consistent with those previously reported in several other meta-analyses [109, 123–125]. In their meta-analysis, the meta-analysis by Taioli et al. showed that colorectal cancers with *MTHFR 677TT* polymorphism was associated with a reduction in CRC risk (corrected OR = 0.85, 95% CI: 0.75, 0.96) [123]. The meta-analysis by Hubner et al. showed an overall superiority ratio of 0.83 (95% CI: 0.75, 0.93) for CRC associated with the *MTHFR 677TT* genotype, which was also in high agreement with the results of the present study [124]. Huang et al. investigated the role of *MTHFR C677T* and *A1298C* polymorphisms in colorectal adenomas and colorectal cancers, and it showed a small but significant negative correlation between CRC and *TT* genotypes (OR 0.93, 95% CI: 0.89, 0.97), which was in agreement with the results of our study [125].

The consistency between our meta-analysis results and the above-mentioned meta-analysis results seems to indicate that MTHFR polymorphisms have variability in CRC prevalence. TT genotype may be a protective factor for colorectal cancer. Combined with the folate metabolism mechanism studied by previous studies, we speculate that the TT genotype may reduce the activity of MTHFR, resulting in a reduced efficiency of converting 5,10-methylene thf into 5-methylene thf. This process ultimately accumulates 5,10-methylenetetrahydrofuran, which promotes the efficiency of DNA synthesis. Of course, this mechanism is a conjecture on our part, and subsequent experiments in related basic disciplines need to be designed for more accurate demonstration. And this speculation is not completely absolute. Due to the multi-factorial influence of colorectal cancer, we believe that this may only be part of the reason why patients with the TT genotype have a reduced risk of colorectal cancer.

### The relationship between folate and the development of colorectal cancer

Studies have shown that supplementation with adequate folate has a positive preventive effect on colorectal adenomas, with a more pronounced effect in patients with inflammatory bowel disease [126]. It was reported that high intake of adequate folate reduces the risk of colorectal cancer [127]. Adequate folate maintains genome stability by regulating DNA biosynthesis, repair, and methylation. Currently, there are two possible mechanisms for the anticancer effects of FA, i.e., the carbon source of DNA synthesis and the regulatory mechanism of DNA methylation [128]. (1) FA is a carbon source for the initial synthesis of nucleotides, and FA deficiency reduces the source of precursors for nucleotide synthesis, leading to DNA damage and ultimately to an imbalance in the nucleotide pool; (2) This imbalance leads to misincorporation of uracil into DNA, as well as DNA mismatches and double-strand breaks. As a result, when normal cells have low levels of FA, the cells accumulate DNA damage and are thus more susceptible to malignant transformation, and therefore proper FA supplementation may reduce the risk of colorectal cancer.

Our results indicate that when folate intake is increased,the incidence of colorectal cancer may be reduced in all three MTHFR genotypes, especially in the *TT* genotype. However, the current research on folic acid intake and colorectal cancer lacks unified standards and is small in number. This makes this conclusion of considerable value, but cannot draw a strong conclusion. It is necessary to enrich folic acid supplements and colorectal cancer. Clinical studies on the association with cancer risk and research on related mechanisms. Secondly, there are many types of folic acid supplements currently on the market. For example, active folic acid in the supplement options for pregnant women has better effects than ordinary folic acid. However, there are few studies on the relationship between active folic acid intake and colorectal cancer. More studies are expected to explore the comparative role of active folic acid and ordinary folic acid intake in the occurrence and development of colorectal cancer.

### Limitations

Regarding the relationship between race, geographical location, even dietary habits and other factors, and folic acid intake and colorectal cancer, most current studies have not clearly considered whether regional dietary differences lead to regional differences in intake. The differences in folic acid ultimately lead to differences in the incidence of colorectal cancer. If this is confirmed, the correlation between folic acid and colorectum will be more powerfully concluded. Of course, due to the complexity of the occurrence and development of colorectal cancer, the above-mentioned regional differences are also likely to be caused by differences in human genes. Perhaps the intake of folic acid in some areas is of little significance. These considerations will ultimately require a large number of studies in the future. The experimental design takes into account the influence of relevant factors. There are currently no in-depth and clear results on the mechanism of folic acid metabolism in the occurrence and development of tumors. Our research is based on the basic role of folic acid and its key enzymes in DNA synthesis and methylation in the body, and we speculate that folic acid may There is a correlation with the incidence of colorectal cancer, and based on all the data searched, a conclusion is drawn that there is a possible correlation. But this is not enough to draw a strong conclusion that folic acid supplements are effective and that MTHFR gene detection is related to predicting the occurrence of colorectal cancer. It is expected that more research on the mechanism in this area will provide more powerful evidence of the role of folic acid in the occurrence and metastasis of colorectal cancer.

## Conclusions and study recommendations

Our analysis supports the conclusion that purity of the *T* allele (*MTHFR 677TT* polymorphism) is associated with a reduced risk of colorectal cancer and based on the impact of this geographic and ethnic variation. We suggest that this association may not apply to all populations. Moreover, folate is also a widely used supplement, so exploring the relationship between folate dose and *MTHFR* gene polymorphisms and the risk of colorectal cancer becomes a complex study that requires a large number of books to exclude the effect of supplements and to standardize the effect of folate intake. Other studies have explored that supplementation with multivitamins, such as folate, negatively correlates with the risk of familial colorectal cancer, so exploring the effect of multivitamins on the risk of colorectal cancer is also a very interesting direction. In conclusion, exploring the association of the *MTHFR C677T* polymorphism with behavioral and environmental determinants of colorectal cancer risk as well as human ethnicity is necessary to better study the risk of colorectal cancer. *MTHFR C677T,* in combination with dietary factors, intake of multivitamins such as folate, and smoking and drinking habits, may constitute a composite risk factor for colorectal cancer that warrants further investigation. These findings require larger studies to elucidate the role of the *MTHFR 677TT* polymorphism and to assess the gene-environment interactions of MTHFR.

## Supplementary Information

Supplementary Material 1: Supplementary Fig. 1. The forest plot of meta-analysis odds ratios for colorectal cancer among persons with the *MTHFR 677CT* and *677CC *genotype in Random effects model.

Supplementary Material 2: Supplementary Fig. 2. The forest plot of meta-analysis odds ratios for colorectal cancer among persons with the* MTHFR 677TT* and *677CC* genotype in Random effects model.

Supplementary Material 3: Supplementary Fig. 3. The forest plot of meta-analysis odds ratios for colorectal cancer among persons with the *MTHFR 677T* genotype with the enhanced intake of folate in Random effects model. The patients were divided into two groups: high and low intakes of folate. The high group was more than the low group at least 100μg/day. (A) The forest plot of meta-analysis of the association between CRC susceptibility and intake of folate. (B) The forest plot of meta-analysis of the association between CRC susceptibility and intake of folate among the* MTHFR 677TT* genotype. (C) The forest plot of meta-analysis of the association between CRC susceptibility and intake of folate among the *MTHFR 677CC*+*CT* genotype.

Supplementary Material 4. 

Supplementary Material 5.

Supplementary Material 6: Supplementary Table 1. The results of quality assessment of elected studies using the Newcastle–Ottawa scale.

Supplementary Material 7: Supplementary Table 2. Relation Between *MTHFR C677T* Genotype and Colorectal Cancer: General Characteristics of Studies verified by random effect model Included in a Meta-Analysis.

## Data Availability

No datasets were generated or analysed during the current study.
