# Supplementary figures and images for: Meta Analysis of Methylenetetrahydrofolate Reductase (MTHFR) C677T polymorphism and its association with folate and colorectal cancer
Source: BMC Cancer. 2025 Jan 29;25:169. doi: 10.1186/s12885-025-13546-w (PMC11776141; doi:10.1186/s12885-025-13546-w)

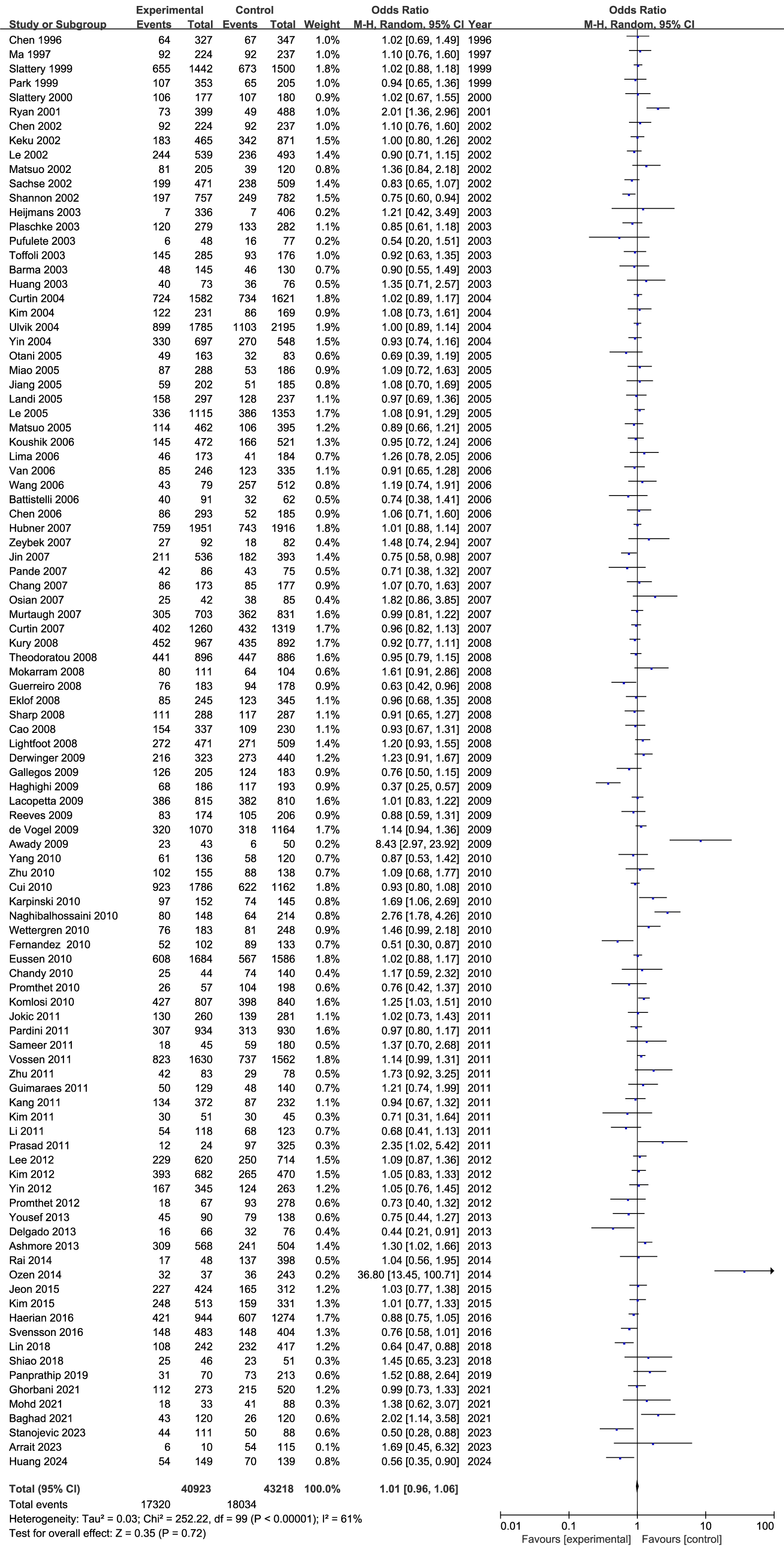

Supplement: Supplementary file 1 — Supplementary Material 1: Supplementary Fig. 1. The forest plot of meta-analysis odds ratios for colorectal cancer among persons with the MTHFR 677CT and 677CC genotype in Random effects model. [file 12885_2025_13546_MOESM1_ESM.tif]

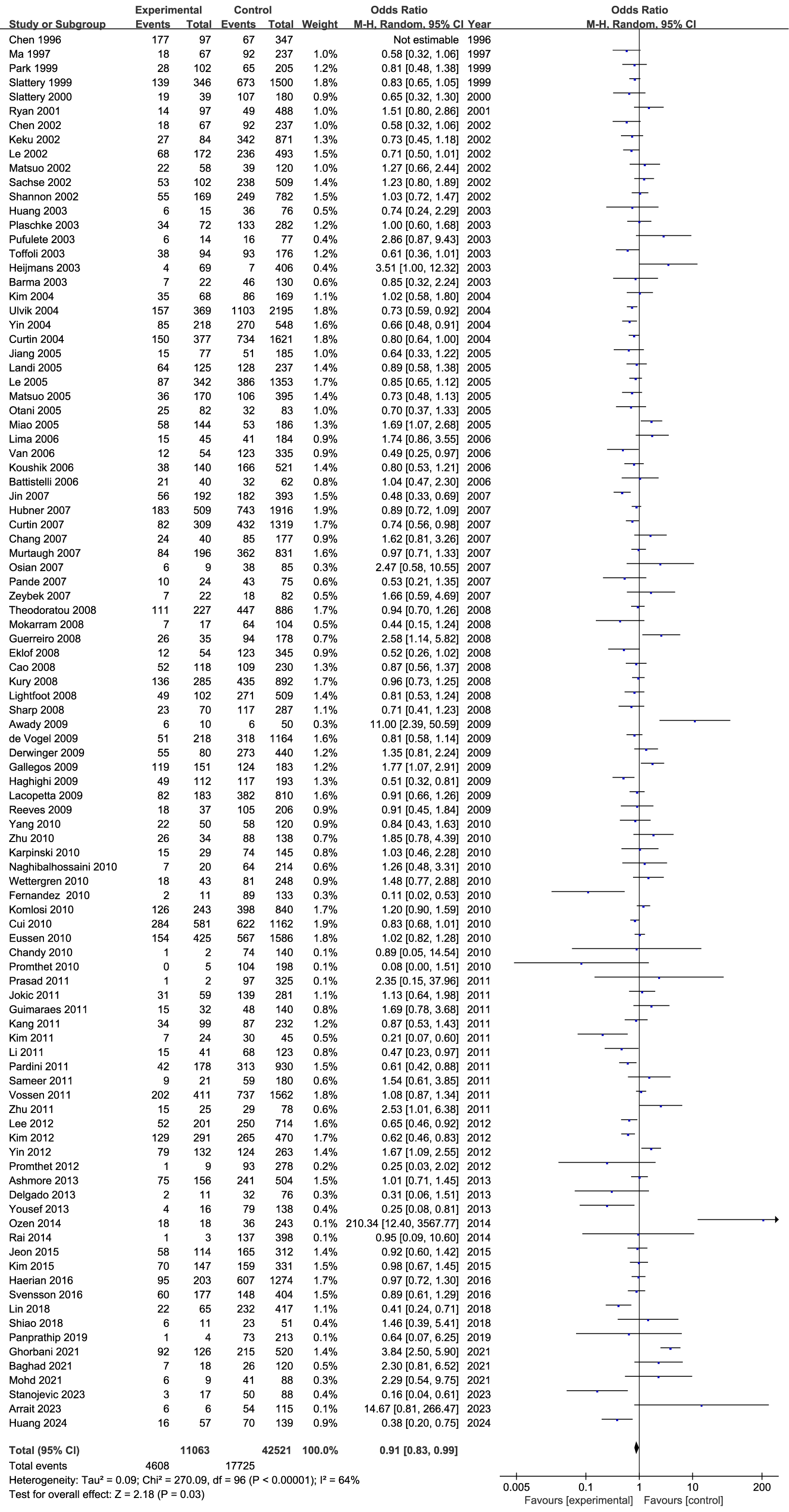

Supplement: Supplementary file 2 — Supplementary Material 2: Supplementary Fig. 2. The forest plot of meta-analysis odds ratios for colorectal cancer among persons with the MTHFR 677TT and 677CC genotype in Random effects model. [file 12885_2025_13546_MOESM2_ESM.tif]

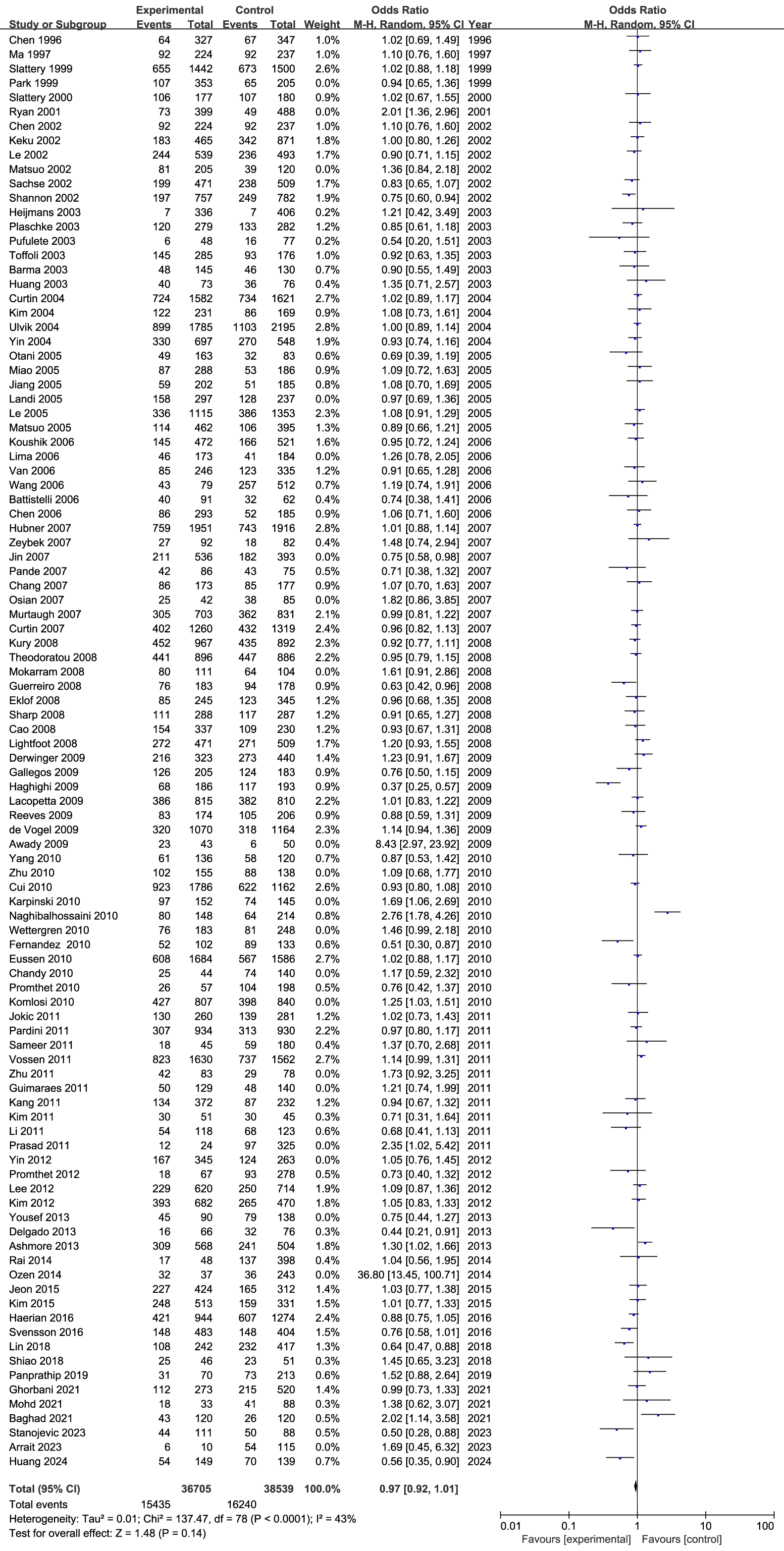

Supplement: Supplementary file 3 — Supplementary Material 3: Supplementary Fig. 3. The forest plot of meta-analysis odds ratios for colorectal cancer among persons with the MTHFR 677T genotype with the enhanced intake of folate in Random effects model. The patients were divided into two groups: high and low intakes of folate. The high group was more than the low group at least 100μg/day. (A) The forest plot of meta-analysis of the association between CRC susceptibility and intake of folate. (B) The forest plot of meta-analysis of the association between CRC susceptibility and intake of folate among the MTHFR 677TT genotype. (C) The forest plot of meta-analysis of the association between CRC susceptibility and intake of folate among the MTHFR 677CC+CT genotype. [file 12885_2025_13546_MOESM3_ESM.tif]

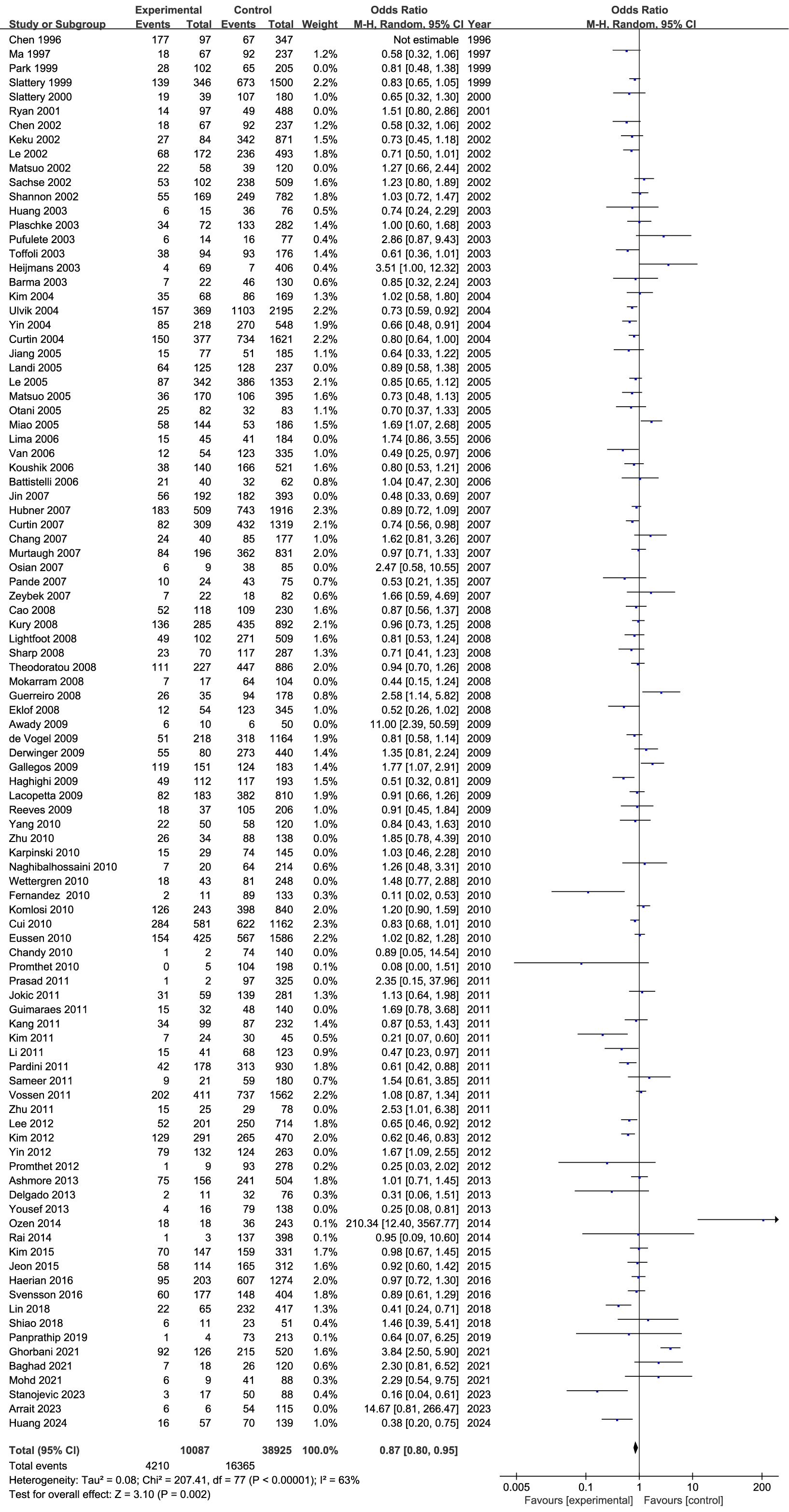

Supplement: Supplementary file 4 — Supplementary Material 4. [file 12885_2025_13546_MOESM4_ESM.tif]

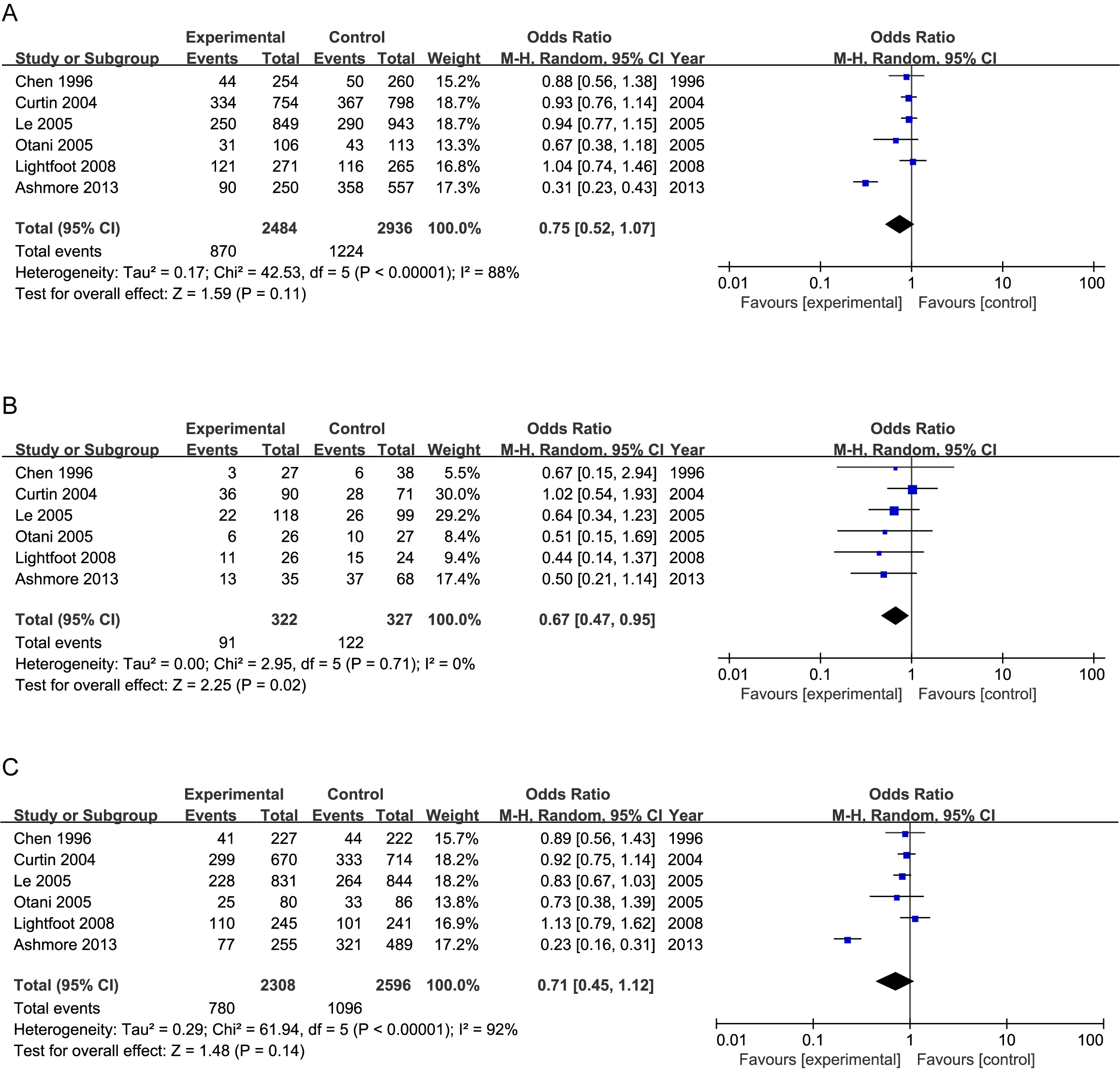

Supplement: Supplementary file 5 — Supplementary Material 5. [file 12885_2025_13546_MOESM5_ESM.tif]

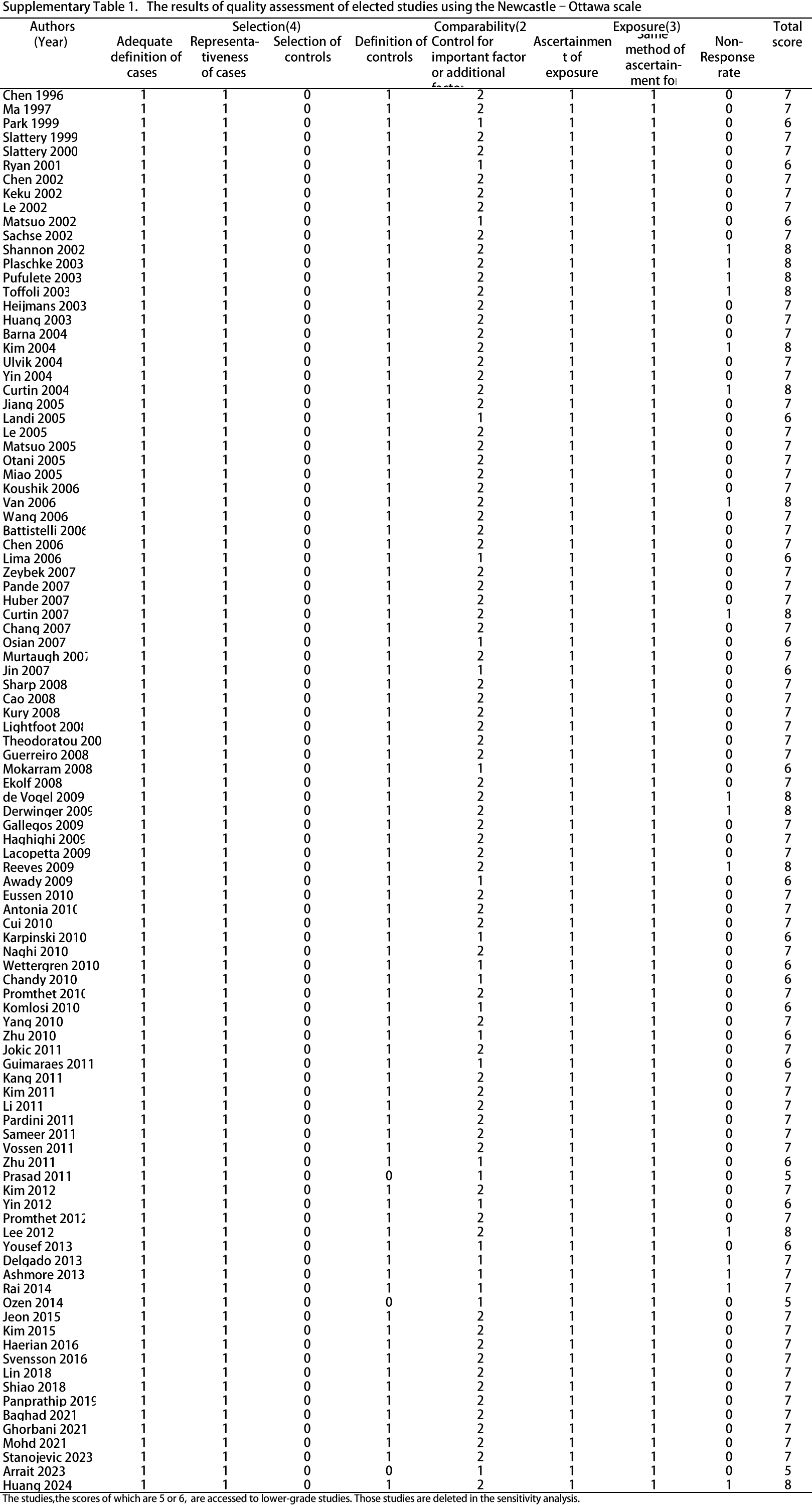

Supplement: Supplementary file 6 — Supplementary Material 6: Supplementary Table 1. The results of quality assessment of elected studies using the Newcastle–Ottawa scale. [file 12885_2025_13546_MOESM6_ESM.tif]

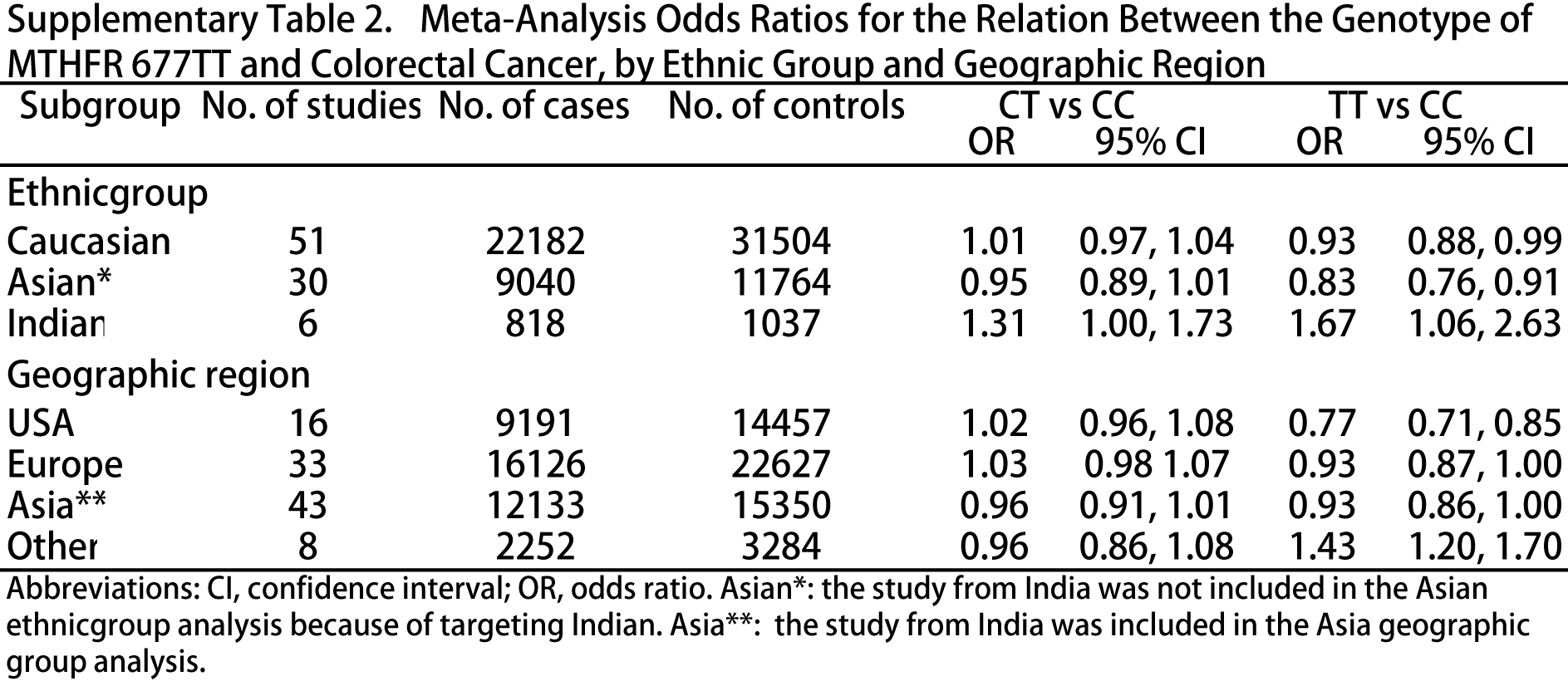

Supplement: Supplementary file 7 — Supplementary Material 7: Supplementary Table 2. Relation Between MTHFR C677T Genotype and Colorectal Cancer: General Characteristics of Studies verified by random effect model Included in a Meta-Analysis. [file 12885_2025_13546_MOESM7_ESM.tif]
